# Molecular detection of *Toxoplasma gondii* and *Neospora caninum* in seabirds collected along the coast of Santa Catarina, Brazil

**DOI:** 10.1590/S1984-29612024019

**Published:** 2024-04-22

**Authors:** Ana Paula Sato, Tiffany Christiny Emmerich da Silva, Thamires Pires de Pontes, Aline Luiza Konell, Luiz Daniel de Barros, Mary Suzan Varaschin, Ivam Moreira de Oliveira, Adrien Wilhelm Dilger Sanches, Rosangela Locatelli-Dittrich

**Affiliations:** 1 Departmento de Medicina Veterinária, Universidade Federal do Paraná - UFPR, Curitiba, PR, Brasil; 2 Projeto de Monitoramento de Praias da Bacia de Santos - PMP-BS, Unidade de Estabilização de Aves Marinhas, Universidade do Vale do Itajaí - UNIVALI, Penha, SC, Brasil; 3 Refúgio Ecológico Bela Vista, Foz do Iguaçu, PR, Brasil; 4 Laboratório de Parasitologia Veterinária e Doenças Parasitárias, Departamento de Medicina Veterinária, Universidade Federal de Lavras - UFLA, Lavras, MG, Brasil; 5 Laboratório de Patologia Veterinária, Departamento de Medicina Veterinária, Universidade Federal de Lavras - UFLA, Lavras, MG, Brasil; 6 Veterinário Autônomo, Curitiba, PR, Brasil

**Keywords:** **:** Marine environment, seabirds, neosporosis, toxoplasmosis, Ambiente marinho, aves marinhas, neosporose, toxoplasmose

## Abstract

*Toxoplasma gondii* and *Neospora caninum* are two closely related protozoans that infect a wide range of animals, including birds. However, the occurrence of *N. caninum* and *T. gondii* in seabirds is unknown. Therefore, this study aimed to determine the presence of *T*. *gondii* and *N*. *caninum* DNA in tissue samples of seabirds. Tissue samples of the pectoral muscles, heart, and brain were collected from 47 birds along the coastline of Santa Catarina State, SC, Brazil. The DNA was extracted from the tissues and screened using nested-PCR (nPCR) targeting internal transcribed spacer 1 (ITS1). *T. gondii* DNA was detected in tissues from seven seabirds (7/47, 14.8%), kelp gull (*Larus dominicanus*) (5/21), and Manx shearwater (*Puffinus puffinus*) (2/8). *N. caninum* DNA was detected in tissues of nine seabirds (9/47, 19.1%), the kelp gull (*L. dominicanus*) (4/21), Manx shearwater (*P. puffinus*) (2/8), neotropic cormorant (*Phalacrocorax brasilianus*) (1/4), brown booby (*Sula leucogaster*) (1/5), and white-chinned petrel (*Procellaria aequinoctialis*) (1/1); however, no co-infection was observed. In conclusion, this study showed the circulation of *N. caninum* and *T. gondii* in seabirds along the coastline of Santa Catarina State. Further studies are required to clarify the role of these birds in the epidemiology of neosporosis and toxoplasmosis.

## Resumo

*Toxoplasma gondii* e *Neospora caninum* são dois protozoários intimamente relacionados que infectam uma ampla gama de animais, incluindo-se aves. Entretanto, a ocorrência de *N. caninum* e *T. gondii* em aves marinhas é desconhecida. Portanto, este estudo objetivou determinar a presença de DNA de *T. gondii* e *N. caninum* em amostras de tecidos, coletadas de 47 aves marinhas. Amostras de tecidos dos músculos peitorais, coração e cérebro foram coletadas de 47 aves marinhas mortas ao longo da costa do estado de Santa Catarina, SC, Brasil. O DNA foi extraído dos tecidos e testado, usando-se a reação em cadeia da polimerase nested (nPCR), tendo em vista o espaçador interno transcrito 1 (ITS1). O DNA de *T. gondii* foi detectado em tecidos de sete aves marinhas (7/47, 14,8%), como gaivota (*Larus dominicanu*s) (5/21) e bobo-pequeno (*Puffinus puffinus*) (2/8). O DNA de *N. caninum* foi detectado em tecidos de nove aves marinhas (9/47, 19,1%); gaivota (*L. dominicanus*) (4/21), bobo-pequeno (*P. puffinus*) (2/8), corvo-marinho (*Phalacrocorax brasilianus*) 1/4), atobá-pardo (*Sula leucogaster*) (1/5), e pardela-preta (*Procellaria aequinoctialis*) (1/1). No entanto, não foi observada coinfecção. Em conclusão, este estudo demonstrou a circulação de *N. caninum* e *T. gondii* em aves marinhas ao longo da costa de Santa Catarina, Brasil. Mais estudos são necessários para esclarecer o papel dessas aves na epidemiologia da neosporose e da toxoplasmose*.*

## Introduction

Seabirds are a diverse group of species that have adapted to marine environments, including coastal areas, estuaries, islands, coastal wetlands, and open sea areas (Schreiber & Burger, 2001). These birds are predators and considered ecological sentinels for environmental health because they feed over large geographic areas at different trophic levels (Parsons et al., 2008; Votier & Sherley, 2017).

The Apicomplexa protozoans, *Toxoplasma gondii* and *Neospora caninum*, are two closely related coccidian parasites that form cysts and are linked to important diseases in animals (Donahoe et al., 2015). Birds can be infected by consuming food and water contaminated with sporulated oocysts of *T. gondii* and/or *N. caninum* and by eating cysts in infected tissues (Barros et al., 2018).

Toxoplasmosis is a common parasitic zoonotic disease caused by *T. gondii*. Felids are the definitive hosts of *T. gondii*; they shed oocysts into the environment, which serve as a source of infection for other animals (Dubey, 2021). Due to the zoonotic potential, the *T. gondii* infection is a public health concern, which could result in congenital disease and other clinically severe presentations in immune-compromised humans (Dubey et al., 2021). Birds are important intermediate hosts of this protozoan and are a source of infection for other animals that consume them (Cabezón et al., 2011). Toxoplasmosis has been documented in domestic and wild avian species, with acute and chronic infection cases reported (Dubey, 2021). However, there is limited data regarding toxoplasmosis in seabird populations (Campbell et al., 2022).

As a waterborne parasite, *T. gondii* has been recognized as a threat to some species of marine mammals in North America, such as sea otters, dolphins, and whales (Jones & Dubey, 2010; Dubey et al., 2020). Studies on marine mammals have shown that environmental oocysts play an important role in the epidemiology of *T. gondii* (Cabezón et al., 2016; Dubey et al., 2020). Sporulated oocysts can be transported to fresh and marine waters via sewage or stormwater drainage systems and freshwater runoff (Conrad et al., 2005). Furthermore, molecular epidemiological surveys have revealed that freshwater can transport *T. gondii* oocysts from terrestrial to coastal marine habitats (VanWormer et al., 2014).

Neosporosis is an infectious disease caused by *N. caninum*, an obligate intracellular Apicomplexan parasite, and is a major cause of bovine abortion worldwide (Donahoe et al., 2015; Barros et al., 2018). Domestic dogs (*Canis lupus familiaris*) and some species of wild canids, such as the Australian dingo (*Canis lupus dingo*), coyote (*Canis latrans*), and gray wolf (*Canis lupus*), are the definitive hosts of this parasite (Cerqueira-Cézar et al., 2017) and several mammals are intermediate hosts (Donahoe et al., 2015). In recent years, birds have been extensively investigated as potential intermediate hosts of *N. caninum* (Barros et al., 2018), with many serological and molecular studies conducted on domestic and wild bird populations (Gondim et al., 2010; Mineo et al., 2011; Darwich et al., 2012; Molina-López et al., 2012; Barros et al., 2017); however, the role of birds in the life cycle of *N. caninum* is not fully understood.

Information on *N. caninum* infection in marine animals is limited, and few molecular studies have been conducted. A previous study showed its exposure in otters, walruses, and dolphins (Dubey et al., 2003). However, the source of these infections is still unclear, and further studies are required to understand better the life cycle of *N. caninum* in marine environments (Fujii et al., 2007; Villagra-Blanco et al., 2019).

Previous studies evaluating *T. gondii* and *N. caninum* in domestic animals (goats, pigs, chickens, cats, and cattle) have been carried out in the Santa Catarina state (Moura et al., 2009; Rosa et al., 2010; Pena et al., 2018; Silva et al., 2020; Costa et al., 2022). The prevalence of anti-*T.gondii* antibodies in cats and dogs from two cities in Santa Catarina were 26% in dogs and 14% in cats (Moura et al., 2009; Rosa et al., 2010). In these studies, access to the street was a risk factor for infection, indicating the circulation of the parasite in the urban center. However, studies to determine the status of these protozoa in wild birds in the state of Santa Catarina are scarce.

Although seabirds are ecological sentinels of environmental health, and Brazil hosts a large proportion of these birds, data on the occurrence of *T. gondii* and *N. caninum* is scarce. Therefore, this study aimed to determine the presence of *T. gondii* and *N. caninum* DNA in seabirds along the Santa Catarina coast, Brazil.

## Material and Methods

### Study area and sample collection

**Figure 1:**
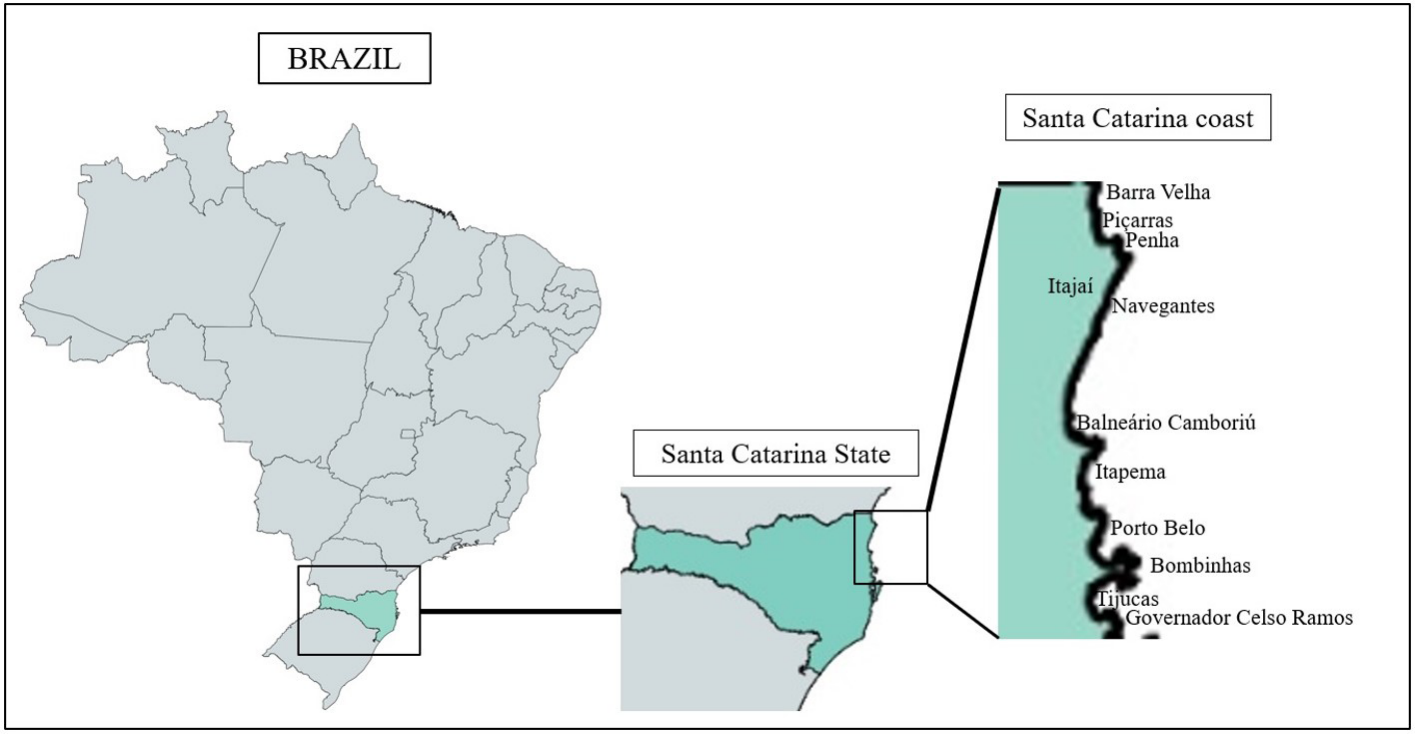
Map of Brazil with the Santa Catarina State coast showing the municipalities where seabirds were collected: Barra Velha, Piçarras, Penha, Navegantes, Itajaí, Balneário Camboriú, Itapema, Porto Belo, Bombinhas, Tijucas e Governador Celso Ramos.

Tissue samples of the pectoral muscles, heart, and brain were collected from 47 dead seabirds from August 2019 to March 2020. The seabirds were collected by the Santos Basin Beach Monitoring Project (PMP-BS) along the Santa Catarina coast, Brazil (Figure 1).

During necropsy, a general examination was performed to determine sex, nutritional status, carcass condition, and gross lesions on the skin, liver, heart, skeletal muscles, respiratory, urinary, and digestive systems, eyes, and encephalon. Taxonomic identification was based on morphologic characters (Harrison, 1989). The age and sex of the seabirds were determined based on plumage and by observing the gonads during the necropsy, respectively (Giaccardi et al., 1997; Griffiths, 2000). Nutritional status was assessed based on the amount of subcutaneous and visceral fat and sternal muscle atrophy. The carcass condition was classified using a four-point system: (1) live animal, (2) fresh/mild decomposition, (3) moderate decomposition, and (4) advanced/severe decomposition (McAloose et al., 2018). Tissue collection was performed by a veterinary pathologist of the PMP-BS, while routine diagnostic protocol was performed to determine the cause of death.

### Tissue digestion and DNA extraction

Pepsin acid digestion was performed on all tissue samples before DNA extraction to increase the number of parasites. Approximately 3 g of each tissue (pectoral muscle, brain, and heart) was macerated separately and digested in 30 mL of digestion solution containing 1.3 g pepsin, 3.5 mL HCl, and 2.5 g NaCl in 500 mL of distilled water (Lindsay et al., 1993). DNA was extracted from 250 µL of digested tissue using a commercial kit (ReliaPrep^™^ gDNA Tissue Miniprep System, Promega Corporation, Madison, WI, USA) following the manufacturer's instructions. Nuclease-free water was included as a negative control in every 15 extraction reactions to ensure no contamination during the DNA extraction process. Extracted and purified DNA was quantified using a spectrophotometer (NanoDrop^™^One, Thermo Fisher Scientific, Waltham, MA, USA).

### Nested-PCR (nPCR)

Nested-PCR (nPCR-ITS1) was performed targeting the 18S and 5.8SrRNA coding genes of the Toxoplasmatinae subfamily (*T. gondii* and *N. caninum*). The forward primers target to the 3’end of the 18S locus, whereas the reverse primers target to the 5’end of the 5.8S locus. The primers flank the complete internal transcribed spacer 1 (ITS-1). JS4 (5’-CGA AAT GGG AAG TTT TGT GAA C-3’) and CT2c (5’-CTG CAA TTC ACA TTG CGT TTC GC-3’) were used as external primers, and JS4b(5’-AGT CGT AAC AAG GTT TCC GTA GG-3’) and CT2b (5-TTG CGC GAG CCA AGA CAT C-3’) were used as internal primers, as previously described (Šlapeta et al., 2002; Soares et al., 2011).

Each amplification was performed in 25 µL reaction mixtures containing 80 ng of DNA, 1.5 mM MgCl_2_, 0.2 mM dNTPs, 1U of Platinum Taq DNA polymerase (Thermo Fisher Scientific), 1 × PCR buffer, and 0.6 µM of each primer and ultrapure water. Reactions were performed using a PCR Thermal Cycler 2720 (Applied Biosystems, Foster City, CA, USA). The same concentration of the abovementioned reagents was used for the second amplification reaction with 1 µL of the product from the first reaction. The cycling conditions for both reactions were as follows: 3 min cycle at 94 °C followed by 35 cycles at 94 °C for 45 s, 56 °C for 30 s, 72 °C for 30 s, and a final extension step at 72 °C for 5 min. The expected amplicon was approximately 500 bp, and positive DNA control samples from *T. gondii* (RH strain), *N. caninum* (NC-1 strain), and the negative control (ultrapure water) were included in all reactions.

The amplified products were subjected to electrophoresis on a 1.5% agarose gel at 90 v for 90 min, stained with SYBR^™^ Safe DNA Gel Stain (Thermo Fisher Scientific), and it was observed using an ultraviolet transilluminator. A 100 bp DNA ladder (Ludwig Biotec, Alvorada, RS, Brazil) was included in all agarose gels.

### Sequencing and Phylogenetic analysis

The obtained PCR products were enzymatically cleaned using a commercial kit following the manufacturer's instructions (ExoSAP-IT^™^ Express PCR Product Cleanup Reagent, Thermo Fisher Scientific). The purified PCR products were sequenced using the BigDye^™^ Terminator v3.1 Cycle Sequencing Kit (Thermo Fisher Scientific) and the internal forward and reverse primers in a Genetic Analyzer 3500xL (Thermo Fisher Scientific), using 50 cm capillaries with a Pop7 polymer. Sequences were analyzed using Chromas and Geneious Prime 2020.1 software. The consensus sequences were compared using the Basic Local Alignment Search Tool (BLAST) with sequences deposited in GenBank at the National Center for Biotechnology Information (NCBI).

The phylogenetic analysis used the maximum likelihood method and the Tamura 3-parameter nucleotide substitution model (Tamura & Kumar, 2002). The phylogenetic tree was constructed using the MEGA11 software, and the bootstrap with 1000 replications was used to test the robustness of the obtained phylogeny.

## Results

Regarding the body condition, of the 47 seabirds, 25 birds scored well, 10 scored poorly, and 12 were cachectic. The carcass condition of 45 birds was classified as 2 (fresh/mild decomposition), whereas two seabirds were classified as 3 (moderate decomposition). The main cause of death in these birds was trauma, followed by asphyxia and cachexia. Nine species included in the study are listed on the IUCN Red List of Threatened Species as “least concern” and one species, the white-chinned petrel (*Procellaria aequinoctialis*), is listed as vulnerable. The seabird species used in this study are listed in Table 1.

**Table 1:** Number of species, taxonomic orders, age, and sex of seabirds found along the coast of Santa Catarina, Brazil, examined in this study.

**Order Common name (species)**	**Number of birds (n)**	**Age**	**Sex**
**Charadriiforme**			
Kelp gull (*Larus dominicanus*)	7	Adult	Male
Kelp gull (*Larus dominicanus*)	6	Adult	Female
Kelp gull (*Larus dominicanus*)	5	Juvenile	Female
Kelp gull (*Larus dominicanus*)	3	Juvenile	Male
Black skimmer (*Rynchops niger*)	1	Juvenile	Male
South American tern (*Sterna hirundinacea*)	1	Adult	Female
Common tern (*Sterna hirundo****)***	1	Adult	Male
Great skua (*Stercorarius skua*)	1	Adult	Female
**Suliformes**			
Neotropic cormorant (*Phalacrocorax brasilianus)*	2	Juvenile	Male
Neotropic cormorant (*Phalacrocorax brasilianus)*	2	Juvenile	Female
Magnificent frigatebird*(Fregata magnificens)*	1	Adult	Male
Brown booby (*Sula leucogaster*)	2	Adult	Male
Brown booby (*Sula leucogaster*)	1	Adult	Female
Brown booby (*Sula leucogaster*)	1	Juvenile	Female
Brown booby (*Sula leucogaster*)	1	Juvenile	Male
**Procellariiformes**			
White-chinned petrel *(Procellaria aequinoctialis)*	1	Adult	Female
Manx shearwater (*Puffinus puffinus*)	6	Juvenile	Female
Manx shearwater (*Puffinus puffinus*)	2	Juvenile	Male
**Sphenisciformes**			
Magellanic penguin (*Spheniscus magellanicus*)	1	Adult	Male
Magellanic penguin (*Spheniscus magellanicus*)	2	Juvenile	Male

The nPCR results were positive for *T. gondii* in 14.8% (7/47) of seabirds and *N. caninum* in 19.1% (9/47). *T. gondii* DNA was detected in tissues from 23.8% (5/21) kelp gull (*Larus dominicanus*) and 25% (2/8) Manx shearwater (*Puffinus puffinus*). *N. caninum* DNA was detected in tissues from 23.8% (821) kelp gulls (*L. dominicanus*), 25% (2/8) Manx shearwater (*P. puffinus*), 25% (1/4) neotropic cormorant (*Phalacrocorax brasilianus*) , 20% (1/4) brown booby (*Sula leucogaster*), and 100% (1/1) white-chinned petrel (*Procellaria aequinoctialis*). No co-infection was observed in all positive animals.

*T. gondii* DNA was detected in the pectoral muscles, heart, and brain samples at 6.38% (3/47), 8.51% (4/47), and 6.38% (3/47), respectively, from seven seabirds (two species). *N. caninum* DNA was detected in the heart, brain, and pectoral muscle samples at 10.6% (5/47), 6.38% (3/47), and 2.12% (1/47), respectively, from eight seabirds (five species). The results of the nPCR are shown in Table 2.

**Table 2:** Results of nested-PCR (nPCR) for *T. gondii* and *N. caninum* in tissues from seabirds found along the coast of Santa Catarina, Brazil.

Common name (species)	nPCR *T. gondii*				nPCR *N. caninum*			
	PM^*^	H^**^	B^***^	Sequence	PM^*^	H^**^	B^***^	Sequence
kelp gull (*L. dominicanus*)	-	-	+	MW021174	-	-	-	
kelp gull (*L. dominicanus*)	+	-	+	MW021175	-	-	**-**	
				MW021507				
kelp gull (*L. dominicanus*)	+	+	+	MW021176	-	-	-	
				MW021421				
				MW021508				
kelp gull (*L*. *dominicanus*)	-	+	-	MW021420	-	-	-	
kelp gull (*L. dominicanus*)	-	+	-	MW021422	-	-	-	
kelp gull (*L. dominicanus*)	-	-	-		+	-	-	MW044668
kelp gull (*L. dominicanus*)	-	-	-		-	+	-	MW022526
kelp gull (*L. dominicanus*)	-	-	-		-	+	-	MW022527
kelp gull (*L. dominicanus*)	-	-	-		-	+	-	MW022528
manx shearwater (*P. puffinus)*	-	+	-	MW023594	-	-	-	
manx shearwater (*P. puffinus)*	+	-	-	MW023595	-	-	-	
manx shearwater (*P. puffinus)*	-	-	-		-	-	+	MW023245
manx shearwater (*P. puffinus)*	-	-	-		-	+	-	MW023247
neotropic cormorant (*P. brasilianus)*	-	-	-		-	-	+	MW044666
brown booby (*S. leucogaster)*	-	-	-		-	-	+	MW044667
white-chinned Petrel (*P. aequinoctialis)*	-	-	-		-	+	-	MW023246

PM*: Pectoral muscle, H**: heart, B***: Brain.

**Figure 2:**
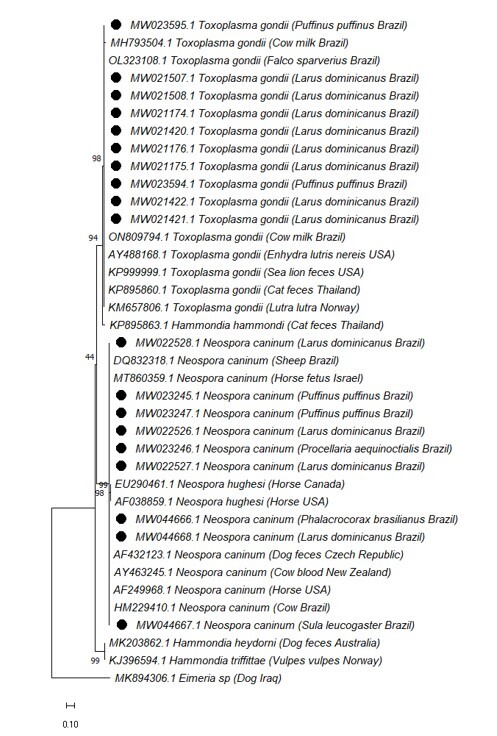
The maximum likelihood phylogenetic tree based on ITS1 sequences. Bootstrap values (1000 replicates) are displayed next to the branches. The black circle indicates the sequence derived from this study. The host, the sample, the strain or isolate name, the country of origin, and the GenBank accession number are shown. *Eimeria* sp. served as an outgroup. The scale bar represented 0.10 changes per nucleotide.

The ITS1 nucleotide sequence data generated in the present study were deposited in GenBank (accession No.: MW021174 - MW021176, MW021420 - MW021422, MW021507 - MW021508, MW023594 - MW 023595, MW022526 - MW022528, MW023245 - MW023247, MW044666 - MW044668) and shared 98-100% identity with *T. gondii* and *N. caninum* sequences. The phylogenetic tree showed that our sequences were clustered with *T. gondii* and *N. caninum* isolates (Figure 2).

## Discussion

In this study, 16 of 47 (34%) seabirds had positive nPCR results, 7 for *T. gondii* and 9 for *N. caninum*, which suggests that seabirds are susceptible to infection with these protozoans. There are few reports of tissue cyst-forming coccidia in seabird species in South America. Thus, to the best of our knowledge, this is the first report of the presence of *T. gondii* and *N. caninum* in the kelp gull (*L. dominicanus*) and Manx shearwater (*P. puffinus*), and *N. caninum* in the neotropic cormorant (*P. brasilianus*), brown booby (*S. leucogaster*), and white-chinned petrel (*P. aequinoctialis*).

There are few studies on DNA detection of these protozoans in seabirds; however, seroepidemiological studies in Spain (Cabezón et al., 2016), Italy (Nardoni et al., 2019a), the Western Indian Ocean (Poulle et al., 2021), and Brazil (Gennari et al., 2016) have shown that these birds are exposed to *T. gondii*. Previous studies have reported the presence of *T. gondii* antibodies in some gull species in Europe, the Western Indian Ocean, and China, with a prevalence ranging from 0-21% (Miao et al., 2014; Cabezón et al., 2016; Poulle et al., 2021). In Brazil, antibodies against *T. gondii* were detected in 24 (34.8%) of 69 seabirds, with titers ranging from 5 to 640 (Gennari et al., 2016).

In the present study, *T. gondii* DNA was detected in seven (14.8%) seabirds, and N. caninum DNA was detected in nine (19.1%). Previous studies reported different DNA detection values for these protozoans in wild birds. Darwich et al. (2012) investigated brain tissue samples from 201 wild birds of 14 species, and detected *T. gondii* and *N. caninum* DNA in 6% and 1.5% of the birds, respectively. Lukášová et al. (2018) investigated the presence of *T. gondii* and *N. caninum* DNA in the brain tissue of 110 wild and domestic birds in Africa. They found three birds (2.7%) positive for *T. gondii* and none positive for *N. caninum*. *Toxoplasma gondii* was isolated from tissues of 16.4% (10/61) of naturally infected black-headed gulls (*Larus ridibundus*) in the Czech Republic (Dubey, 2002). These variations in the detection of *T. gondii* and *N. caninum* DNA in different studies may be attributed to different environmental and/or geographic conditions, bird species tested, molecular methods used, the sample size, and the tissue analyzed (Cabezón et al., 2016; Lukášová et al., 2018).

In the present study, the number of seabirds infected with *N. caninum* was higher than that of birds infected with *T. gondii*. Previous data on the occurrence of *N. caninum* antibodies in wild birds showed variations from 0-34% between species; waterfowl had the highest seroprevalence (Nardoni et al., 2019b), and raptors and geese had the lowest (Rocchigiani et al., 2017; Konell et al., 2019; Sato et al., 2021). Studies have suggested that susceptibility to *N. caninum* infection in birds may be species-specific, with birds of prey appearing to be more resistant. In contrast, passerines, pigeons, and waterfowl appear to be more susceptible to infection (Rocchigiani et al., 2017; De Barros et al., 2018). However, the role of the seabirds in the distribution of *N. caninum* to definitive hosts is unknown and needs further studies to elucidate.

Feeding habits play an important role in determining the levels of exposure of bird species to protozoans (Wilson et al., 2020). Seabirds are predators at risk of horizontal acquisition of infection through the consumption of infected prey harboring tissue cysts or through ingesting water or food contaminated with sporulated oocysts (Cabezón et al., 2016). The contamination of coastal areas with sewage and runoff from freshwater, which may carry *T. gondii* oocysts, is considered an important factor in the epidemiology of toxoplasmosis in the marine environment (Shapiro et al., 2019). Molecular epidemiological studies have provided evidence that *T. gondii* oocysts are transported to marine ecosystems through sewage and freshwater runoff and remain infectious for up to 24 months (Marino et al., 2019; Shapiro et al., 2019). In aquatic ecosystems, oocysts accumulate in bivalve mollusks and fish by filtration and consumption (Lindsay et al., 2004; Massie et al., 2010), respectively, which has been suggested as a route of transmission because these fish and mollusks are a source of food for seabirds and marine mammals.

Our data showed that the kelp gulls (*L. dominicanus*) were the most affected seabird species. This result indicates exposure to *T. gondii* and *N. caninum* in this seabird species. The kelp gull (*L. dominicanus*) belongs to the order Charadriiformes and is the most abundant seabird on the southern coast of Brazil (Soares & Schiefler, 1995; Branco, 2003). The increase in the population of kelp gulls is attributed to the availability of food found in garbage of human origin discarded from fishing (Barbieri, 2008). This species is considered one of the most opportunistic and generalist species among seabirds. Investigating the transmission patterns of *T. gondii* and *N. caninum* in wild birds, especially in those exhibiting opportunistic and generalist feeding behaviors, is important for understanding the epidemiological role of these birds in the maintenance and dissemination of protozoan parasites (Cabezón et al., 2016).

Notably, beach sand is also a source of infection besides seawater contamination with oocysts. Although beach sand can act as a reservoir for various microorganisms, few epidemiological studies have verified the prevalence of *T. gondii* and *N. caninum* (Brandão et al., 2020; Valério et al., 2022). *Toxoplasma gondii* and *N. caninum* oocysts can contaminate beach sand after infected felids and canids shed the parasite in their feces (Donahoe et al., 2015; Shapiro et al., 2019). Oocysts tend to concentrate at or near the defecation sites of cats and dogs but can be dispersed by wind, arthropods, and rainfall (Afonso et al., 2007).

In the present study, the DNA of *T. gondii* and *N. caninum* was detectable in the three tissues analyzed; however, the heart was the tissue with the highest detection rate. Previous studies have reported *T. gondii* tropism in the brain tissue, liver, and heart (Sharma et al., 2019; Campbell et al., 2022). Based on these findings, the heart could be considered a good target for Apicomplexa DNA detection in seabirds.

*T. gondii* and *N. caninum* nucleotide sequences from this study were similar to *T. gondii* and *N. caninum* sequences isolated from various hosts in different geographic regions. A limitation of the present study is that we only evaluated the ITS1 region using primers that amplify other coccidian members of the *Sarcocystidae* family (*T. gondii*, *N. caninum*, *Hammondia heydoni,* and *Sarcocystis* spp.). Although some studies have shown that the ITS1 region is a good marker for establishing differences between closely related coccidia (Šlapeta et al., 2002), sequencing does not allow the detection of any possible co-infections between the two pathogens.

The results of this study indicate the circulation of these protozoans in the Brazilian seacoast and indicate that seabirds could play an epidemiological role in the cycle life of *T. gondii* and *N.caninum*. Seabirds are considered sentinels of environmental contamination due to their ecological position in the trophic chain (Parsons et al., 2008). In addition, seabirds can also serve as a source of infection for felids and dogs that consume them (Doherty et al., 2017; Mendoza Roldan & Otranto, 2023).

## Conclusions

Seabirds are susceptible to infection with *T. gondii* and *N. caninum*. This study detected *T. gondii* and *N. caninum* DNA in kelp gull (*L. dominicanus*) and in Manx shearwater (*P. puffinus*), and only DNA from *N. caninum*in the neotropic cormorant (*P. brasilianus*), brown booby (*S. leucogaster*) and white-chinned petrel (*P. aequinoctialis*). Further investigations are required to clarify the role of seabirds in the life cycle and the epidemiology of these protozoans.
